# Description of the male of *Tityus kuryi* Lourenço, 1997 and notes about males of *Tityus stigmurus* (Thorell, 1877) and *Tityus serrulatus* Lutz & Mello, 1922 (Scorpiones, Buthidae)

**DOI:** 10.3897/zookeys.435.6694

**Published:** 2014-08-15

**Authors:** Maria Dulcinéia Sales dos Santos, Tiago Jordão Porto, Rejâne Maria Lira-da-Silva, Tania Kobler Brazil

**Affiliations:** 1 Universidade Federal da Bahia, Departamento de Zoologia, Instituto de Biologia, Núcleo Regional de Ofiologia e Animais Peçonhentos. Rua Barão de Jeremoabo, 147, Salvador, Bahia, Brazil, 41170-115; 2 Escola Bahiana de Medicina e Saúde Pública. Av. Dom João VI, n°274, Brotas. Salvador, Bahia, Brazil, 40290-000

**Keywords:** Sexual population, scorpions, *Tityus*, Brazil

## Abstract

The male of *Tityus kuryi* Lourenço, 1997 is described for the first time. Despite being very similar to the female, the male presents more robust metasomal segments. Additionally, the distribution of the sexual populations of another two species of the *T. stigmurus* complex is reported herein: *T. serrulatus* Lutz & Mello, 1922 and *T. stigmurus* (Thorell, 1877). Males of *T. serrulatus* were, until now, restricted to the Minas Gerais State (Southwestern region of Brazil), and with new records reported here, its known distribution now encompasses the Northeastern region of Brazil. Males of *T. stigmurus* were previously recorded only for two municipalities in the State of Bahia, and here we present eight new records for Bahia State and one for Pernambuco State. We present a key to related species of the *T. stigmurus* complex based on morphology and coloration pattern.

## Introduction

The description of males in population of scorpions is an important contribution, not only as regards taxonomic knowledge of the species, but also to enable understanding of its reproductive strategy. *Tityus kuryi* Lourenço, 1997 was described based on a single adult female collected in Palmeiras Municipality, in the Chapada Diamantina region, Bahia State, Brazil (Lourenço 1997). Although this species has previously been described, the male has not been described yet. In the last taxonomic review, Souza et al. (2009) included *Tityus kuryi* in the *Tityus stigmurus* complex, together with other five related species: *Tityus aba* Candido, Lucas, Souza, Diaz & Lira-da-Silva, 2005; *Tityus martinpaechi* Lourenço, 2001; *Tityus melici* Lourenço, 2003; *Tityus serrulatus* Lutz & Mello, 1922 and *Tityus stigmurus* (Thorell, 1887). This was due to the fact that they share the spinoid granules of the posterior dorsal region of the metasomal segments III and IV (Souza et al. 2009).

The two more widely distributed species from the *Tityus stigmurus* complex are parthenogenetic: *Tityus serrulatus* and *Tityus stigmurus* (Matthiessen 1962, Ross 2010). Both species are considered scorpions of medical importance (Brasil 2009). Despite their recognized asexual reproductive strategy, especially observed in urban areas, sexual populations have been recognized in Brazil, where males were described (Souza et al. 2009). Males of these two species were first reported by Lourenço and Cloudsley-Thompson in 1999 stating sexual populations in Minas Gerais and Pernambuco states. In the last review of the *Tityus stigmurus* complex, Souza et al. (2009) redescribed the males of *Tityus serrulatus* and *Tityus stigmurus* with greater number of details, especially in relation to dimorphism. These last authors based their study on specimens of *Tityus serrulatus* from Espinosa Municipality, Minas Gerais State, and on specimens of *Tityus stigmurus* from Camaçari and Paulo Afonso Municipalities, Bahia State. As far as we know, these are the only published records of males from both species.

In this paper, we describe the male of *Tityus kuryi* and report new records of *Tityus serrulatus* and *Tityus stigmurus* males, widening the known distribution of their sexual populations.

## Materials and methods

Specimens of *Tityus kuryi* (n = 9), *Tityus serrulatus* (n = 1595) and *Tityus stigmurus* (n = 380) present in the scientific collection of the “Museu de Zoologia da Universidade Federal da Bahia (MZUFBA)” were analyzed. Along with these, another 280 specimens of *Tityus stigmurus* present in the reference collection of the “Centro de Informações Anti-veneno do Estado da Bahia” (CIAVE), Health Department of Bahia State, were also studied. Of the nine *Tityus kuryi* examined, four of them were kept in captivity for a year and a half (August 2009 to February 2011), a fact that allowed us to identify the spermatophore and to confirm the presence of two males.

The measurements were obtained following the methodology of Sissom et al. (1990) and using a digital Starrett 727 caliper. Trichobothrial notations follow Vachon (1974) while morphological terminology mostly follows Hjelle (1990), except for pedipalp carinae (Stahnke 1970). Observations of the morphology were made using a LEICA Z4 stereomicroscope. The photos of the specimens were taken with a Nikon D7000 camera, Micro Nikkor 85mm and Micro Nikkor 105mm lenses. The photos and measurements of the hemispermatophore were taken using the software program Motic Images 2000 version 1.2 by means of a PC connected to a Motic Digital stereomicroscope SMZ 168. The map of geographic distribution was produced with the ArcGis 10.0 software.

The males of *Tityus serrulatus* and *Tityus stigmurus* were identified based on observation of the external morphological characteristics, as diagnosed by Souza et al. (2009). The identification of the male of *Tityus kuryi* was based on observation of the courtship behavior, copulation and deposition of spermatophore (MZUFBA 2569), carried out in captivity occurred in May 2010. The hemispermatophore here described was dissected and deposited in the arachnid collection (MZUFBA 2570).

### Abbreviations

MZUFBA Museu de Zoologia da Universidade Federal da Bahia (Zoology Museum of the Federal University of Bahia), Salvador, Bahia, Brazil.

CIAVE Centro de Informações Anti-veneno do Estado da Bahia (Anti-Poison Information Center of Bahia), Salvador, Bahia, Brazil.

## Taxonomy

### Family Buthidae C. L. Koch, 1837
Genus *Tityus* C. L. Koch, 1836

#### Tityus kuryi

Taxon classificationAnimaliaScorpionesButhidae

Lourenço, 1997

**Figures 1–4.**
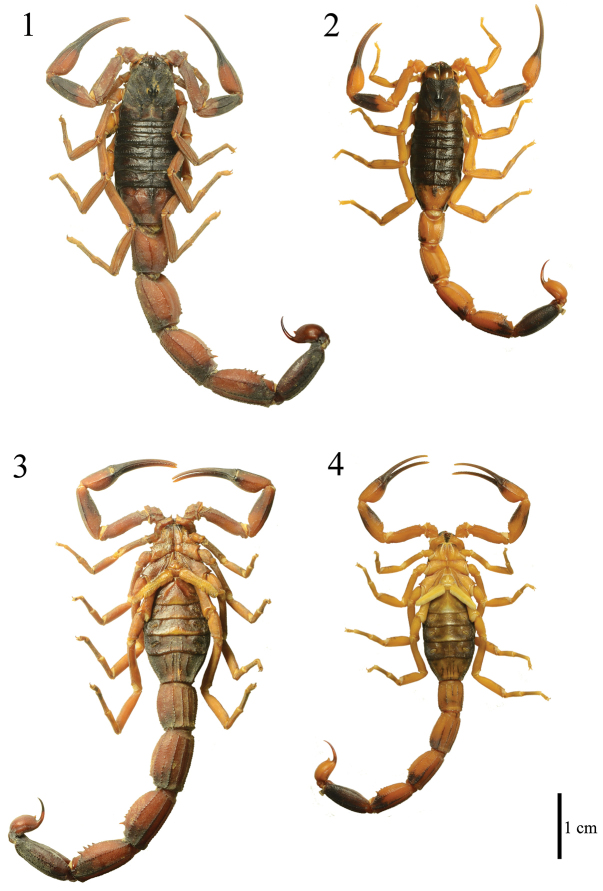
*Tityus kuryi*. Male (MZUFBA 2569 - Palmeiras, Bahia, Brasil): dorsal (**1**) and ventral (**3**) views. Mature female (MZUFBA 2451 - Ibicoara, Bahia, Brazil): dorsal (**2**) and ventral (**4**) views.

Figs 1 [Fig F2] [Fig F3] –14 ; Table 1

##### Material examined.

Brazil, Bahia State, Palmeiras Municipality, Vale do Capão, 12°37'04"S, 41°29'20"W, 850 m, 24/XII/2006 (T.J. Porto leg.), adult male (MZUFBA 2569); Brazil, Bahia State, Palmeiras Municipality, Vale do Capão, 12°37'11"S, 41°29'23"W, 850 m, 24/XII/2006 (T.J. Porto leg.), adult male (MZUFBA 2570); Brazil, Bahia State, Palmeiras Municipality, Vale do Capão and Cachoeira da Fumaça, 12°31'44"S, 41°33'32"W, 04/VI/1999, 23/II/2007 and 17/VII/2009 (C. M. Pinto-Leite & G. Carvalho leg.), five adult females (MZUFBA 1000, 1602, 2166, 2505, 2529); Brazil, Bahia State, Ibicoara Municipality, 13°24'4"S, 41°16'5"W, I/2005 and VII/ 2011, two adult females (MZUFBA 2451, 2572).

##### Diagnosis.

Scorpion of medium to large size, ranging from 55 to 78mm in total length. General coloration dark reddish with blackish spots on the pedipalps, legs, lateral surfaces of mesosomal tergites and ventral submedian carinae of all metasomal segments, as well as transversal blackish spots on the posterior margin of sternites. Carinae moderately to strongly marked; granulations moderately to weakly marked. Fixed and movable fingers with 16–17 oblique rows of granules. Pectines with 24–25 teeth in males, 23–26 in female. Secondary sexual dimorphism evident.

##### Comparisons with related species.

*Tityus kuryi* Lourenço, 1997, belongs to the “*Tityus stigmurus*” species complex. The male of *Tityus kuryi* can be distinguished from the other males of the species complex, particular from *Tityus aba*, *Tityus stigmurus* and *Tityus martinpaechi*, by the absence of three longitudinal dark brown stripes on mesosomal tergites. Furthermore, in *Tityus aba* and *Tityus martinpaechi*, the pedipalp of the males is much thinner than of females, which also occurs in *Tityus melici* although there is no metasomal dimorfism in it (Souza et al. 2006, Souza et al. 2009). *Tityus kuryi* show pedipalps with no dimorphism (Figs 1–4). However, the male metasoma is more robust than the female, the same pattern of differentiation that can be observed in other species of the *Tityus stigmurus* complex, such as *Tityus serrulatus* and *Tityus stigmurus* (Souza et al. 2009).

##### Key to related species of the *Tityus stigmurus* complex

**Table:** 

1	Metasomal segments III and IV without or with 1 to 3 granules modified as spines	2
−	Metasomal segments III and IV with 5 to 7 granules modified as spines	5
2	Longitudinal dark stripes over tergites present	3
−	Tergites densely pigmented, without longitudinal stripes	*Tityus melici*
3	One longitudinal dark stripe evident over tergites	*Tityus stigmurus*
−	Three longitudinal dark stripe evident over tergites	4
4	Prosoma predominantly dark, pedipalps and legs without spots, pectines with 25–25 teeth	*Tityus aba*
−	Prosoma with dark inverted triangle, pedipalps and legs pigmented, pectines with 23–23 teeth	*Tityus martinpaechi*
5	Coloration dark reddish, pedipalps and legs pigmented	*Tityus kuryi*
−	Coloration yellowish, pedipalps and legs without spots	*Tityus serrulatus*

##### Description.

Based on male MZUFBA 2569.

**Figures 5–11. F2:**
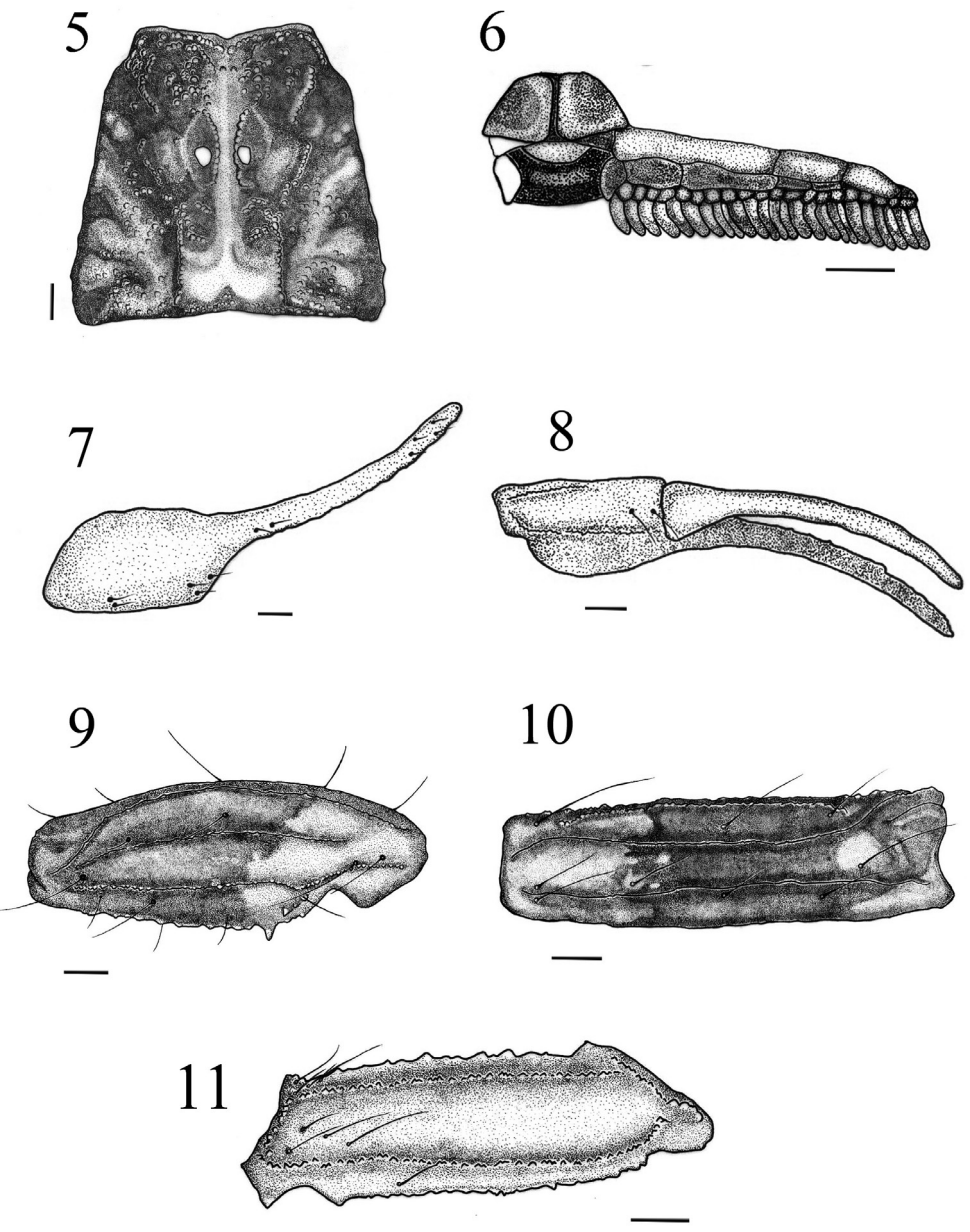
*Tityus kuryi* (male). **5** Carapace **6** Pectines **7–8** Chela, dorsal external and ventral views **9–10** Patella, dorsal and external views **11** Femur, dorsal view. Scale bars= 1 mm.

**Figure 12. F3:**
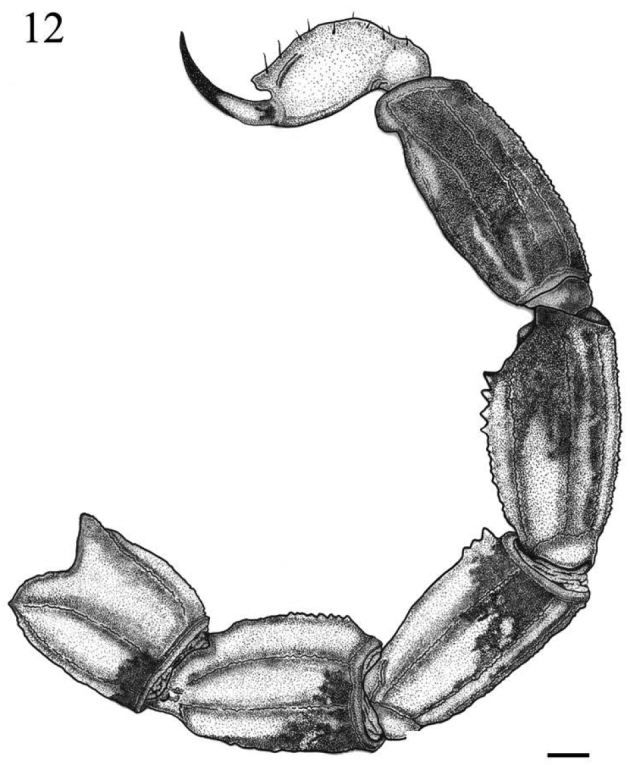
*Tityus kuryi* (male): Lateral views of the metasoma and telson. Scale bars= 1 mm.

*Coloration*: Reddish brown with numerous dark areas (Fig. 1). Carapace dark with some light-brown areas (Fig. 5). Ocular tubercle dark. Mesosoma dark in tergite VI, tergite VII with a darker central region and lighter red-brown lateral region (Fig. 1); metasomal segments I–IV reddish brown with dark areas posteriorly in the lateral region and on the submedian ventral carinae (Figs 1 and 3); a dark spot occupying almost the entire segment V (Fig. 12). Telson: vesicle reddish brown, lighter than the metasomal segment V, with two small spots at the base; aculeus with dark spots at the base, red-brown medially and blackened distally (Fig. 12). Coxosternal region yellow with black spots in the coxapophyse I and II (Fig. 3); sternite III light brown, sternites IV–VI darker with the posterior medial region light brown, sternite VII darker with medially light brown T-shaped spots (Fig. 3). Chelicerae dark with a light brown base; apex of the fingers brown. Pedipalps reddish brown with dark spots in the patella (Figs 9–10) and chela (Figs 7–8); fingers generally dark but distally light brown. Legs light brown with dark spots on tibia and tarsi (Fig. 1).

*Morphology*: Carapace: anterior margin with a weak median concavity (Fig. 5); median ocular tubercle situated anterior to the center of the carapace and median eyes separated by more than one ocular diameter. Three pairs of lateral eyes; median ocular carina moderate with medium granules (Fig. 5); anterior median furrow moderately deep. Moderately granular.

*Mesosoma*: Tergites moderately granular with larger granules in the posterior region; presence of median carinae in all tergites. Tergites I–II with reduced carinae; in the tergites III–IV the carinae occupy the distal half and begin bifurcated and finish merged; tergite VII pentacarinate; transversal carinae present in all tergites. Pectines: pectinal teeth 24–25, basal middle lamellae of pectines not dilated (Fig. 6). Sternites weakly granular; a clear triangular zone in the posterior region of the sternite III and a reduced smooth and shiny slightly expanded triangular zone in the posterior region of the sternite V (Fig. 3). Sternite VI with two short median carinae occupying the distal half. Sternite VII with five carinae, two parallel submedian, occupying the entire sternite with a small carinae between them, and two lateral carinae restricted to the central region.

*Metasoma*: Metasomal segments: I with ten complete paired carinae (ventral submedian, ventral lateral, lateral inframedian, lateral supramedian and dorsal lateral with adjacent granules, the dorsal lateral has one spinoid posterior granule) (Fig. 12); II with eight complete paired carinae (inframedian lateral carinae incomplete on anterior third and present sparse granules; others are complete with adjacent granules; dorsal lateral with one spinoid granule) (Fig. 12); III with eight complete paired carinae (inframedian lateral carinae absent; others complete with adjacent granules, the dorsal lateral with three spinoid posterior granules) (Fig. 12); IV with eight complete paired carinae (inframedian lateral carinae absent; others are complete with adjacent granules; dorsal lateral with four spinoid posterior granules) (Fig. 12); V with five complete carinae with uniform and adjacent granules (inframedian lateral carinae and dorsal lateral carinae absent; two complete paired carinae: ventral submedian and ventral lateral; one ventral median carina); intercarinal surface moderately granular (Fig. 12). Telson: vesicle with five carinae (four of which vestigial and only the ventral well defined); aculeus long and strongly curved; subaculear tooth strong and romboid with two small dorsal teeth (Fig. 12).

*Chelicerae*: Dentition as defined by Vachon (1963) for the family Buthidae.

*Pedipalp*: (Figs 9–11) femur with 5 carinae, the dorsointernal, dorsoexternal and externomedian carinae present median granules; ventrointernal with smaller granules and internomedian with larger granules; patella with 7 carinae, the dorsoexternal, internomedian, ventrointernal and dorsomedian carinae present median and adjacent granules; internomedian with a proximal spinoid granule (Fig. 9); dorsoexternal, externomedian and ventroexternal with small and continuous granules; chela with 9 carinae of which the dorsoexternal, dorsal secondary, dorsointemal, ventrointernal, internomedian, ventroexternal, digital, subdigital and ventromedian, all with small and continuous granules; all pedipalp surfaces moderate to weakly granular. Movable fingers with 17–17 oblique lines of granules. Trichobothriotaxy: ortobothriotaxy A–α (Vachon 1974, 1975).

**Figures 13–14.**
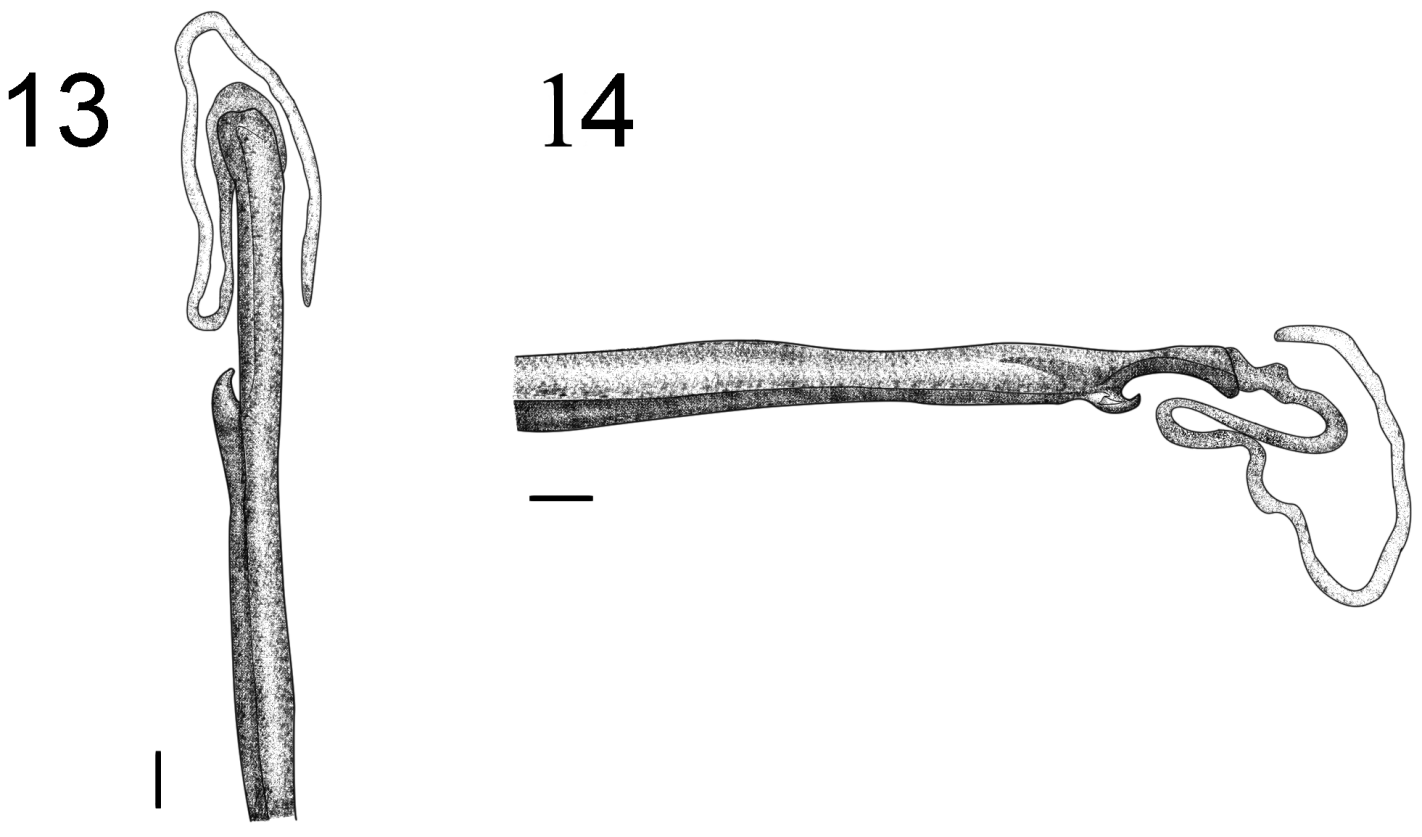
Hemispermatophore of *Tityus kuryi* (male MZUFBA 2570; Palmeiras, Bahia, Brazil): dorsal (**1**) and lateral (**2**) views. Scale bars= 1 mm

*Hemispermatophore*: Flagelliform, long and narrow, measuring approximately 13.5 mm, general color light brown; the trunk is trough-shaped; the flagellum is half the width of the trunk and approximately the same length (Fig. 13). Presence of three distal lobes: basal lobe, internal lobe and external lobe. The basal lobe is hook-shaped, protruding internally or externally; the internal lobe extends up to the base of the basal lobe flagellum, the external lobe extends from the medial basal lobe to the posterior third of the internal lobe (Fig. 14). The basal and external lobes are blackened.

*Sexual dimorphism*: Despite being similar to the females with regard to the coloration pattern and morphological details of the species (Figs 1–4), the male of *Tityus kuryi* presents metasomal segments 1.5 time more robust than those of the females (Figs 1–4; Table 1).

**Table 1.** Measurements (in mm) of two males and seven females used to investigate the sexual dimorphism in *Tityus kuryi*.

	Males		Females						
**MZUFBA number**	**2569**	**2570**	**2451**	**2505**	**2529**	**2572**	**1000**	**1602**	**2166**
**Total Length**	72.9	72.9	60.2	59.2	58.5	60.8	67.6	71.5	78.1
**Carapace**									
Length	8.6	8.5	8.0	6.7	7.2	8.1	8.2	8.1	8.3
Anterior width	5.5	5.9	5.0	5.0	5.2	5.2	4.3	4.6	4.6
Posterior width	7.8	9.1	7.7	6.7	7.4	7.1	9.0	8.8	8.8
**Metasoma**									
Segment I (length)	7.0	7.9	5.9	4.4	5.4	5.2	5.8	5.5	6.0
Segment I (width)	6.9	6.3	4.6	4.1	4.4	4.3	5.0	5.0	4.8
Segment II (length)	9.5	9.1	6.9	6.2	6.5	6.6	6.8	7.1	7.0
Segment II (width)	7.0	6.8	4.6	4.2	4.4	4.5	5.0	5.1	4.8
Segment III (length)	10.2	10.1	7.7	6.8	7.0	8.2	7.5	7.6	7.6
Segment III (width)	7.0	6.	4.9	4.4	4.3	4.7	5.1	5.1	5.0
Segment IV (length)	10.8	10.7	8.7	7.7	7.9	4.6	8.2	8.3	8.1
Segment IV (width)	6.5	6.7	4.6	4.2	4.3	8.2	5.5	5.1	5.0
Segment V (length)	10.6	10.4	8.7	8.4	8.3	9.1	9.5	8.8	9.1
Segment V (width)	5.7	5.8	4.2	4.0	4.1	4.2	4.6	4.1	4.5
**Vesicle**									
Length	8.5	8.7	8.0	5.9	7.3	6.9	6.8	8.2	8.3
Depth	2.5	2.4	2.7	1.5	1.1	2.6	2.7	2.7	2.7
**Pedipalp**									
Femur (length)	8.1	8.4	7.5	6.5	7.6	6.6	8.2	7.6	7.6
Femur (width)	2.2	2.2	2.0	1.8	1.9	1.8	2.2	2.5	2.3
Patella (length)	9.2	9.0	8.5	7.8	7.5	8.4	8.2	8.0	8.2
Patella (width)	2.8	2.9	2.8	2.4	2.8	2.9	3.2	3.2	3.2
Chela (length)	16.2	16.0	14.5	13.4	13.5	14.3	14.3	14.3	14.3
Chela (width)	3.0	3.0	2.9	2.7	2.7	2.9	3.1	3.1	3.1
Movable finger (length)	10.5	9.6	9.6	8.5	8.7	9.2	9.7	9.7	9.7

##### Distribution.

Known only from the Chapada Diamantina region, Bahia State, Brazil.

##### Variation.

The male of *Tityus kuryi* is usually larger than the female (male: 72.93–72.95 mm; female: 58.54–78.03mm) and pectinal tooth counts varied as follows: 24–25 teeth in males and 23–26 in females. The number of principal rows of granules varied from 16–17 in both sexes. The spinoid granules on the posterior end of the dorsal lateral carinae of metasomal segments III and IV are greater in number, is larger and sharper on the left than on the right, and the counts varied as follows: 1–3 in metasomal segment III and 2–4 in metasomal segment IV.

##### Ecology.

*Tityus kuryi* occurs in a restricted environment of montane savannas biome named “Campos Rupestres” of the Espinhaço Range (Cadeia do Espinhaço), in rocky areas of high altitudes in the Chapada Diamantina, Bahia State. The annual average temperature there is 22–24 °C, with 36–38 °C as absolute maximum and 4.8 °C as absolute minimum. It is found at altitudes above 840 m. They can also be found near to residential areas, but always near to natural fragments hidden in debris under or between stones.

#### Distribution of *Tityus serrulatus* and *Tityus stigmurus* males

In an attempt to explain the strategies of the life history in populations of scorpions, Vandel (1928 *apud* Lourenço 2008) was the first to use the term “geographical parthenogenetics” to name the features that various authors had already observed: the parthenogenetics and sexual populations of the same species tend to occur in different habitats.

The sexual populations of *Tityus serrulatus* and *Tityus stigmurus* have a highly restricted geographic distribution, while asexual populations (parthenogenetic) of both species have a wide geographic distribution, occupying urban areas across the Country (Lourenço 2008). This reproductive strategy is advantageous, it allows rapid colonization and wider dispersion in disturbed environments (Lourenço and Cuellar 1995, Lourenço 2002). Thus, it allows these scorpions to occupy areas where sexual populations have difficulties in establishing colonies (Lynch 1984, Cuellar 1994). This can increase the species reproductive capacity by about twofold, as parthenogenesis is of the thelytoky type (production of all-female progeny) (Glesener and Tilman 1978).

Although *Tityus serrulatus* has been known as parthenogenetic since 1962 (Matthiesen 1962), this not occur with *Tityus stigmurus*, whose confirmation of reproductive strategy came much later when Ross (2010) reported the occurrence of thelytokous parthenogenesis in this species. In both, parthenogenesis is not a mandatory feature, as shown in last review of the *Tityus stigmurus* complex that describes males for both species (Souza et al. 2009). The new records that we present in this study (Table 2) indicate the occurrence of sexual populations of *Tityus stigmurus* in eight municipalities of Bahia, in addition to those already known, Camaçari and Paulo Afonso (Souza et al. 2009) and one record in Pernambuco State (see Table 2). These records improve the knowledge on the distribution of the sexual populations of this species.

**Table 2.** New records of males of *Tityus kuryi*, *Tityus serrulatus* and *Tityus stigmurus*.

Species	Registration number	Municipality and state of Brazil	Latitude (DMS)	Longitude (DMS)
*Tityus kuryi*	MZUFBA 2569	Palmeiras, Bahia	12°31'44"S, 41°33'32"W	
	MZUFBA 2570	Palmeiras, Bahia	12°31'44"S, 41°33'32"W	
*Tityus serrulatus*	MZUFBA 2573	São Desedério, Bahia	12°35'21"S, 44°98'42"W	
*Tityus stigmurus*	MZUFBA 2339	Exú, Pernambuco	07°30'43"S, 39°43'26"W	
	MZUFBA 166	Santo Estevão, Bahia	12°25'49"S, 39°15'05"W	
	MZUFBA 168	Santo Estevão, Bahia	12°25'49"S, 39°15'05"W	
	MZUFBA 270	Ruy Barbosa, Bahia	12°17'02"S, 40°29'38"W	
	MZUFBA 2104	Iraquara, Bahia	12°14'56"S, 41°37'08"W	
	MZUFBA 2297	Morro do Chapéu, Bahia	11°33'00"S, 41°09'21"W	
	MZUFBA 763	Feira de Santana, Bahia	12°16'00"S, 38°58'00"W	
	CIAVE 41	Jacobina, Bahia	11°10'50"S, 40°31'06"W	
	CIAVE 23	Lauro de Freitas, Bahia	12°53'38"S, 38°19'37"W	
	CIAVE 617	Salvador, Bahia	12°58'16"S, 38°30'39"W	
	CIAVE 923	Salvador, Bahia	12°58'16"S, 38°30'39"W	

**Figure 15.**
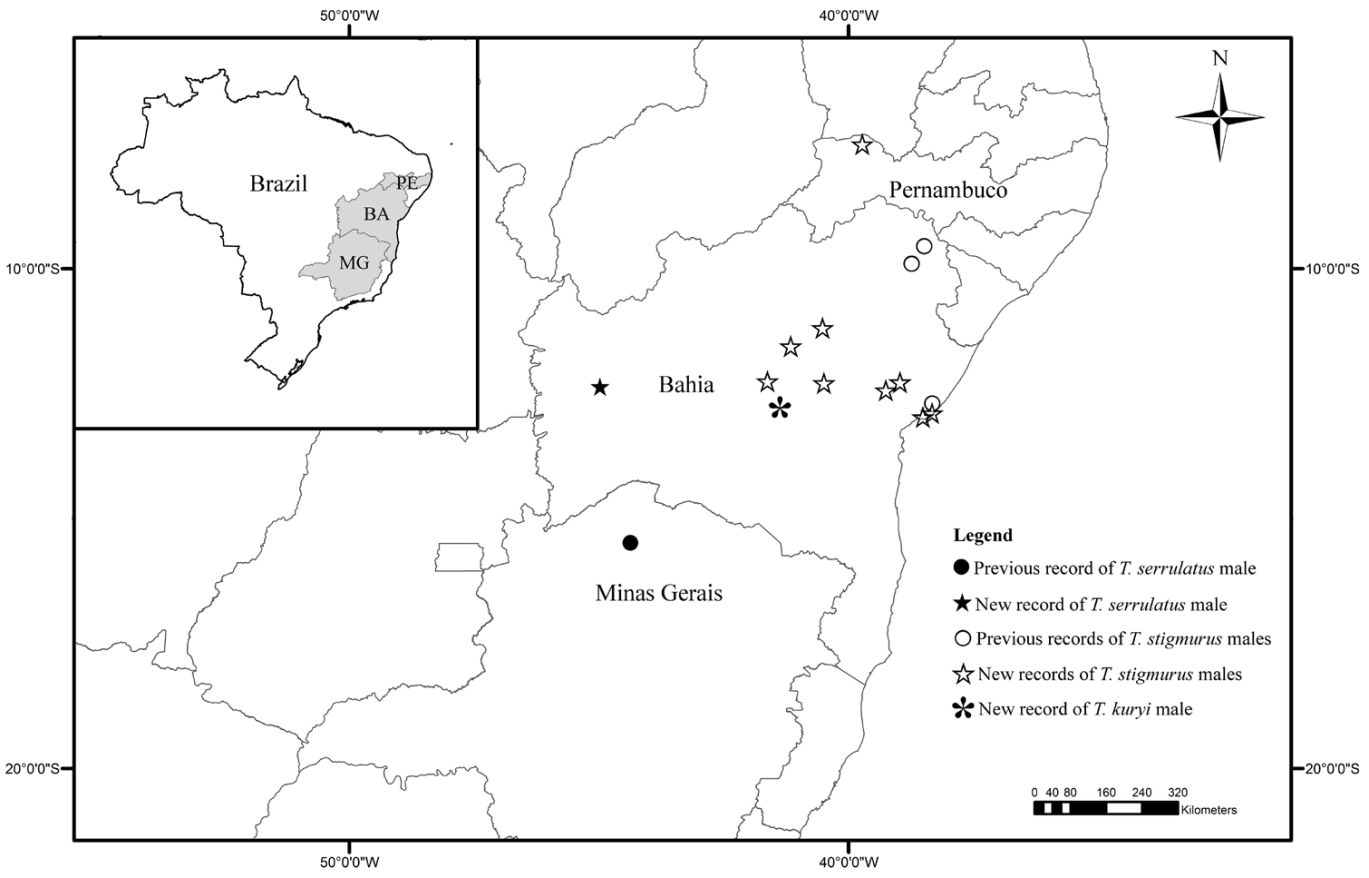
Map showing the previous and new records of males of *Tityus kuryi*, *Tityus serrulatus* and *Tityus stigmurus*.

The male specimen of *Tityus serrulatus* used in this study is deposited in MZUFBA and came from the municipality of São Desiderio, Bahia. We can say now that sexual population of this species, previously restricted to the State of Minas Gerais (municipality Espinosa) and Southeastern Brazil (Lourenço and Cloudsley-Thompson 1999, Souza et al. 2009), can also be found in the Northeast of the Country (Fig. 15).

In spite of the new records of sexual populations of *Tityus serrulatus* and *Tityus stigmurus* to previously unknown areas, parthenogenesis seems to be the main reproductive strategy of these species. This assertion is primarily based on the low frequency of males of these species in three of the largest scorpion collections in Brazil (Instituto Butantan-SP, Museu Nacional-RJ and MZUFBA) and on our field experience collecting with a UV flashlight. Unlike other parthenogenetic scorpions, *Tityus serrulatus* and *Tityus stigmurus* are actually the two species better adapted to urban environments. They are regarded as a public health problem due to their rapid expansion in urban areas, their proliferation and the toxic effects of their poison (Lourenço and Cloudsley-Thompson 1999, Brasil 2009).

We can emphasize here the large number of individuals analyzed as opposed to the few males found, providing evidence of the rarity of males, both in nature and in scientific collections.

## Supplementary Material

XML Treatment for Tityus kuryi
